# Foreign Summary

**Published:** 1839-10-12

**Authors:** 


### FOREIGN SUMMARY.

### Velpeau's Clinical Lectures on Ophthalmia. No. V.

*On the Treatment of Inflammation of the Conjunctiva. Treatment of Conjunctivitis.*

The treatment of conjunctivitis, like that of all other surgical diseases, may be either general or local. The general treatment is regulated by the constitution of the patient, and the symptoms to which the malady may give rise. The local treatment, on the contrary, is directed against the lesion itself. In conjunctivitis, as, indeed, in all the various inflammatory actions we have yet to study, local treatment is more especially to be depended upon. I do not mean to assert that general remedies, such as bleeding, purgatives, &c., should be banished from the treatment of these diseases; you must be well aware that circumstances too numerous to be mentioned render them often useful, and in some cases even necessary. I wish you, however, to understand that the action of these agents is indirect—that they undoubtedly assist in effecting a cure, but in most instances are not sufficient to produce it of themselves. My ideas on this subject have not been formed *à priori*, but are founded on numerous facts and experiments, which have led me to the conclusion that local remedies ought to occupy the first place in the treatment of ophthalmia.

On examining the local agents which we can employ in the treatment of these affections, we shall find that, as in blepharitis, astringent applications are to be preferred. The treatment of the several forms of conjunctivitis, although not essentially different, presents, nevertheless, modifications of sufficient importance to induce me to treat separately the remedies applicable to each.

*Simple Conjunctivitis.*

When the inflammation is slight, you will often find that bathing the eye with some emollient liquid, such as an infusion of marsh mallow or linseed, or even with tepid water alone, will in a few days dissipate every inflammatory symptom. If, however, the inflammation is intense, these means no longer suffice, and you must have recourse to astringent applications, under the form of collyria. The substances generally employed by practitioners are, sulphate of zinc, acetate of lead, or nitrate of silver. The different preparations of the nitrate of silver, which you see me use so frequently, and to which I wish more especially to draw your attention, are by no means modern remedies. The ancients recommend the nitrate of silver, but only as a caustic, in cases of chronic conjunctivitis. Now, you must have observed that in our wards this remedy is principally used as a collyrium or ointment in cases of acute inflammation, and that the success attending this practice is really surprising. Regent, Janin, and Scarpa, who speak in high terms of the nitrate of silver, appear only to have employed it as a caustic. I cannot, however, admit that it acts as a caustic only; its action, in many cases, is certainly resolutive, and the rapidity with which it arrests the progress of inflammation in intense conjunctivitis is a decided proof that I am warranted in making this assertion. You have, indeed, seen numerous instances this year, in which the inflammation has been thus rapidly arrested.

Whatever may be the mode of action of the nitrate of silver collyrium, it deserves the decided preference of practitioners. The experiments I have made to ascertain its efficacy are so numerous, that I have no hesitation in speaking thus decidedly. I must, however, add that it is more especially in simple conjunctivitis that the effects of the nitrate of silver are rapid and undeniable. I generally commence with a solution, containing half a grain to an ounce of distilled water, for children, and a grain or a grain and a half to the same quantity of water for adults; the proportion is afterwards gradually increased. When the solution has been used for a short time, it should be laid aside for two or three days, and then again employed as before; and the treatment should be thus continued until the inflammation is entirely subdued.

During the first few days you employ the collyrium, the inflammatory symptoms are often exasperated, and this might create doubts in your mind respecting the efficacy of the remedy—the more so, as, when its use is suspended, the inflammation diminishes, and the disease appears to progress rapidly towards a favourable termination. The patients themselves, indeed, are generally inclined to think that the treatment they have undergone has done them more harm than good; but a well-informed medical practitioner will never entertain such an opinion, as the above mentioned phenomena are in perfect accordance with the laws of pathology.

The solution must be again employed after a few days' intermission, as I have already stated. Were it to be discontinued, from erroneous views respecting its action, the disease would, in all probability, return with greater intensity even than before.

I must also add, that the efficacy of the nitrate of silver collyrium depends, in a great measure, on the manner in which it is applied, it being absolutely necessary that every portion of the inflamed conjunctiva should be brought into contact with the solution. I need scarcely say that it ought never to be employed as a lotion; two or three drops only should be introduced between the eyelids night and morning. To effect this, the patient must lean his head back, and then, separating the eyelids as far as possible, you allow two or three drops to fall from the phial on the eye. You should then immediately close the eyelids, and keep them in contact for a few seconds, at the same time directing the patient to move the eye in various directions.

I have entered into these details respecting the nitrate of silver collyrium, because it is, without exception, the remedy on which we may place the greatest reliance, not only in the affections which we are now studying, but in several others of which I shall have to speak hereafter. We must not, however, forget, that in many cases local treatment alone is not sufficient, and that we are then obliged to have recourse to general means. Thus, if the inflammation is intense, and the patient of a plethoric habit of body, it becomes necessary to bleed. In some cases of this nature, I have derived great benefit from bleeding *coup sur coup*, according to M. Bouillaud's method. If, on the contrary, there appear to be slight symptoms of gastric irritation, purgatives may be resorted to. In a word, general symptoms, whatever they be, when they exist, should be treated as in all other inflammatory affections.

*Conjunctivitis with Chemosis.*

I have described two forms of chemosis essentially different from one another; you will not, therefore, be surprised to hear that the treatment of each of these forms is also essentially different.

When you meet with the inflammatory or phlegmonous chemosis, you must at once have recourse to antiphlogistics, following up the treatment with greater or less energy, according to the degree of intensity of the inflammation. In these cases, bleeding from the arm *coup sur coup*, and leeches applied to the temples, or to the mastoid region, are often attended with very favourable results. I have in many instances found this practice exceedingly beneficial. Direct applications have also been much employed in this, the acute form of chemosis. From the earliest periods, surgeons have attempted to bring about the resolution of the inflammation by scarifying the injected tissues. The ancients, you well know, performed this operation with barley spikes. I am, however, inclined to think that scarification of the conjunctival vessels does not deserve the praise given to it not only by the ancients, but also in our own times by M. Demours, and by the English practitioners, who, I believe, employ it pretty generally. M. Sanson says he has found scarification in some instances a valuable remedy. It may be so; as, however, my experience on this subject has not been very extensive, I do not feel warranted in giving a decided opinion. I must, at the same time, remind you, that we have in our possession a more efficacious, and certainly less dangerous remedy; I allude to leeches applied to the surface of the conjunctiva itself. In 1817 I made several experiments, at Tours, with M. Bretonneau, to ascertain the effects produced by leeches thus applied; and I must say we always found that the patient derived great benefit from their application.

I do not wish entirely to banish scarifications from the treatment of inflammatory chemosis, as cases may occur in which they ought to be resorted to; but these cases are rare; general blood-letting and leeches, applied as I have stated, are almost always sufficient to subdue the chemosis. I seldom have recourse even to leeches; not that they cause much pain, but from the trouble attending their application. They are not, it is true, long in filling themselves, but they have to be applied one by one, which makes it rather a tedious affair.

To resume, the following is the course I generally pursue. I begin by general and local blood-letting, regulating the quantity of blood extracted, and the number of times the operation is repeated, according to the intensity of the inflammation, and the constitution of the patient. If these general means are not sufficient to calm the inflammatory symptoms, I then apply leeches to the conjunctiva. When the tumefaction and inflammation have subsided, I have recourse to the nitrate of silver collyrium. By thus uniting general and local treatment, I frequently succeed in subduing the chemosis in the course of a few days; and should you adopt this plan in your practice, I am convinced it will be followed by the same results.

The second form of chemosis—the serous or œdematous form—not being essentially of an inflammatory nature, it is no longer necessary to resort to general or local depletion. Purgatives seem to be the most useful remedy we can employ in this affection; and among them calomel, given in Rasorian doses, occupies undoubtedly the first rank. Here again, however, the nitrate of silver collyrium is the agent on which we must chiefly depend for a radical and definitive cure of the disease.

*Partial Conjunctivitis.*

The treatment is the same in this form of inflammation as in simple conjunctivitis. I shall, therefore, confine myself to a caution respecting the use of the nitrate of silver collyrium. It must be employed with rather more circumspection than hitherto, and applied, as far as possible, to the parts only which are inflamed, in order that the remainder of the mucous membrane should not be too much irritated.

*Papular Conjunctivitis.*

The nitrate of silver collyrium does not always prove sufficient to effect the cure of papular conjunctivitis. When this remedy does not succeed, the best plan we can adopt is that of cauterizing lightly the papulæ with the solid nitrate. As soon as this has been done, it is advisable to drop a little cold water over the cauterized spot, with the view of preventing the parts of the conjunctiva you wish to preserve intact, from being injured. During the two or three days which follow this slight operation, the symptoms are exacerbated, the intensity of the inflammation being apparently much increased. You have already seen that this is generally the case, even when the solution is employed. The patient, however, soon begins to feel better, the inflammation diminishes, and, when the papulæ have been cauterized a time or two, entirely disappears. This form of conjunctivitis is much more obstinate than those we have already examined: this is a fact you ought always to bear in mind.

*Granular Conjunctivitis.*

After what I have said respecting the seat and the symptoms of granular conjunctivitis, you will not be surprised to find that it is the form of conjunctivitis which usually proves the most rebellious to therapeutical agents. The disease is not, however, incurable, a well-directed treatment, continued with perseverance, being generally successful,—more frequently, indeed, than in granular blepharitis. The treatment of this affection is exactly the same as that of granular blepharitis. I shall, therefore, to avoid repetition, refer you to what I stated in a former lecture, with regard to the treatmeat of this malady.

These are the considerations I have to offer you respecting the treatment of the various forms of conjunctivitis we have already examined. I shall now proceed to describe the purulent forms of inflammation.

*Purulent Conjunctivitis.*

All the morbid affections described by authors under the name of purulent ophthalmia may be reduced, as regards their primitive seat, to two principal classes:—

1st. Purulent blepharitis; that is, purulent blepharitis of new-born children, or purulent blepharitis of adults.

2d. Purulent conjunctivitis.

I have already stated my reasons for making this division; it would therefore be useless to repeat what I have said.

Purulent conjunctivitis is generally admitted to be contagious. The facts which can be adduced to support this opinion are so numerous, that I scarcely consider it necessary to discuss the question; there can, indeed, be no doubt on the subject. The disease seems often to rage as an epidemic; and there are, perhaps, few countries where, at one period or another, it has not exercised its ravages. There are, consequently, many interesting treatises on this malady to be met with, nearly each epidemic having had its historian. Were you to form an opinion from the mere perusal of these treatises, you would admit numerous forms of purulent conjunctivitis. I am, however, inclined to think that the authors of these monographs, led astray, in some degree, by a minute appreciation of peculiar symptoms, perhaps also influenced by the charm of novelty, have considered the disease they had observed as a new affection, although, in reality, it had no claim to such a distinction. If we examine with care, and without any prevention, the descriptions they give, we cannot but acknowledge that there exists the greatest analogy between the various epidemics; an analogy which warrants my considering them as mere modifications of two principal forms of disease. The forms to which I allude, are—

1. Gonorrhœal conjunctivitis.
2. The purulent conjunctivitis of Egypt.

I do not intend to examine separately the numerous epidemics which have been witnessed and described in our own and other countries; we have not time to devote to such a study. If any of you, however, doubt the correctness of these remarks, I would request you to draw a parallel between the different descriptions; you would then see immediately that these affections are, in reality, nearly identical. If you listen attentively to what I am about to say, you will, I think, be able to form a correct opinion respecting purulent ophthalmia.

*Gonorrhœal Conjunctivitis.*

Although numerous researches have been made to ascertain the nature of this affection, practitioners are far from agreeing with regard to its original mode of production.

Some look upon the disease as the result of a metastasis of the urethral affection to the mucous membrane of the eye; others attribute it to gonorrhœal matter being, by some means or other, brought in contact with the conjunctiva; some, again, attribute it to a general syphilitical infection of the system. Each of these opinions numbers many partisans, who bring forward numerous facts and arguments to defend their theoretical views. Indeed, any one of these opinions might be adopted, without any offence to reason, to the exclusion of the other two. Whatever may be the manner in which gonorrhœal conjunctivitis originates, no one in the present day can doubt its syphilitic nature; the facts which establish this point are too numerous to be denied, and this is quite sufficient to guide us in practice. We will now proceed to describe the symptoms by which the malady may be recognized.

One of the most striking features of this disease is the extreme rapidity with which it progresses. The rapidity is, indeed, so great in some cases, that it becomes impossible to follow the march of the complaint through its different periods. The eye may be lost in less than four-and-twenty hours, and that without its being possible for the surgeon to arrest the progress of the malady. This is not, however, always the case; the march of the disease may be sufficiently slow to enable us to study it in its different stages. These are the cases I shall take as models in the description I am about to give you. In either form, however, the affection is a most serious one, and one in which the surgeon must not temporise, but act at once, and with energy.

When gonorrhœal inflammation of the conjunctiva is moderate, the following are the symptoms which we observe:—The conjunctiva, at first of an uniform red cinnabar colour, soon becomes of a darker hue, and assumes a tinge similar to that of wine-lees. The injected vessels are no longer to be distinguished from one another, and the tissue itself of the membrane, as well as the cellular layers separating it from the sclerotica, becomes gorged with blood, and tumefied. The free surface of the conjunctiva, at first smooth and polished, soon assumes a velvety appearance, and presents a great number of granulations, the volume of which varies from the size of a millet seed to that of a large pea. This tumefaction of the conjunctiva gradually increasing, soon gives rise to chemosis, with all the concomitant symptoms.

The tissues which enter into the formation of the palpebræ are generally more or less inflamed. The eyelids become much swollen, and the cutaneous surface is red and hot, the patient complaining at the same time of burning pain in this region. Sometimes the tumefaction of the upper eyelid is so considerable, as to partly cover the lower one. Sometimes, on the contrary, there is ectropion, and then the velvety appearance of the globe of the eye is perfectly visible; the eye presenting a fungous, granular surface, in the centre of which we see the cornea, more or less concealed by the tumefaction of the conjunctiva. The most important character, however, the character which may be considered pathognomonic of this form of ophthalmia, is to be found in the thick greenish-yellow purulent mucus, which is continually discharged from the eye,—mucus which may be compared in every respect to the discharge we meet with in gonorrhœa. If the eyelids are separated, the purulent fluid which is retained between the eye and the palpebræ by the tumefied state of these organs, flows in a stream over the face. In this affection, as in those we have already examined, the visual functions are not disordered unless the swelling be so great as more or less to conceal the pupil, and thus diminish the field of vision. The deep-seated pain felt by the patient must be attributed to the compression exercised on the globe of the eye by the inflamed and tumefied tissues.

These symptoms, if borne in mind, will always enable you to recognize gonorrhœal conjunctivitis. You must not, however, think that this affection is always met with as I have just described it,—that is, in the simple inflammatory form, without complication. Unfortunately, the inflammation is seldom confined to the conjunctiva. In many instances it attacks the cornea, and it is to this complication I particularly wish to draw your attention. It is not always in our power to prevent the inflammation extending to the cornea, which is then often destroyed in the course of a few hours, although previously perfectly healthy and transparent. In some cases, nevertheless, we are able to follow the progress of the disease after it has reached the cornea. When this is the case, we find that the symptoms are developed with extraordinary rapidity. The cornea loses its transparency, and becomes of a whitish-gray colour. Vision is also necessarily impaired, the patient complaining of a mist which obstructs the sight. The tissue of the cornea seems infiltrated with an opaque fluid; a kind of pultaceous matter covers the free surface, from which it is easily separated. Very soon, pus takes the place of the pultaceous matter, and an ulceration is seen, with perpendicular edges of variable size. This ulceration growing deeper and deeper, the cornea is at last entirely perforated, and the humours of the eye evacuated. The iris then becomes engaged in the perforation of the cornea; and the interior of the eye, which is the seat of intense inflammation, suppurates profusely.

I have often remarked in gonorrhœal conjunctivitis, that when the secretion of mucus is considerable, of a purulent nature, and by its acrid properties gives rise to slight inflammation of the skin over which it passes, the cornea remains perfectly sound; whereas, if the secretion is less abundant, of a whitish colour, of a creamy consistence, and does not irritate the skin, we often find that the cornea is perforated in the manner I described. This circumstance has also been noticed by other practitioners. You must not, however, look upon this as a general law; were you to do so, you might often be led to entertain false hopes with regard to the termination of the disease.

Gonorrhœal conjunctivitis does not always terminate thus unfavourably. An energetic, well-directed treatment will sometimes destroy the inflammation at the onset, and the cure may be thus completed by the local treatment I shall presently describe.

In the most favourable cases, when the disease terminates by resolution, the secretion of mucus becomes every day less thick and less abundant. The tumefaction also diminishes, and by degrees every tissue returns to its normal state. The resolution is, however, seldom complete, the conjunctiva retaining more or less of its vascular appearance, and the mucous follicles remaining voluminous and indurated. A glutinous, half-purulent matter continues to be secreted. When the eye is in this state, the slightest cause will bring back the acute form of inflammation. The surgeon should, therefore, use every endeavour to subdue the inflammation which still exists in this, the subacute form.

Sometimes, after gonorrhœal conjunctivis, the cornea presents opacities which interfere more or less with vision, according to their extent, and according to the part of the cornea they occupy. I merely allude now to these lesions of the cornea, as I shall speak of them at length when treating of keratitis.

*Treatment of Gonorrhœal Conjunctivitis.*

If all I have said respecting this malady, has been well understood, you must, in some measure, be already aware of the nature of the treatment which ought to be pursued. At the very commencement of the disease, as soon as he is aware of its presence, the practitioner must attack it with energy. The least delay on his part, or the inefficacy of the agents he employs, is generally attended with fatal consequences to the organ affected. When the disease is a little farther advanced, it is too late; the most powerful measures are then nearly always unable to arrest the rapid strides of the malady.

Purulent conjunctivitis is undoubtedly the form of ophthalmia which most imperiously requires general treatment. You must, therefore, at once have recourse to blood-letting, general and local. The practice of bleeding *coup sur coup*, to syncope, is often attended with very beneficial effects. I have several times resorted to this mode of treatment, and that with success; the intensity of the inflammation being, indeed, such as to warrant the most energetic measures. When, by copious and repeated bleeding, you have diminished the volume of the circulating fluid, you must endeavour, by the depletion of the inflamed tissues, to act more directly on the malady. The means by which this may be accomplished are various. Some recommend leeches applied round the orbit, on the mastoid region; others, the opening of the temporal artery, or the scarification of the conjunctiva. Arteriotomy does not certainly deserve the praise which has been given to it by some. When other means fail to arrest the progress of the inflammation, I should not expect greater success from such an operation. As, however, it is a plan I have not very often tried, I will not speak very decidedly on the subject.

Excision of a portion of the conjunctiva, which has been recently extolled by M. Sanson, is often attended with serious consequences; we ought, therefore, to be careful how we make use of such a remedy. I should myself prefer repeated applications of leeches on the conjunctiva. By adopting this plan the end proposed is attained, and the disagreeable consequences which may follow the excision are avoided.

When you have subdued the inflammation by general and local depletion, to complete the cure you must have recourse to another plan of treatment. The measures to which I have just alluded are, it is true, indispensable in the great majority of cases. Nevertheless, alone, without the assistance of local applications, they would not restore the inflamed tissues to their normal state. Before I speak of topical remedies, however, I must say a few words respecting a special mode of treatment which has been proposed by some practitioners.

Reasoning on the supposed gonorrhœal nature of the malady, they consider it advisable to recall the discharge from the urethra; this is to be effected by various means. Some propose introducing into the urinary canal a bougie impregnated with gonorrhœal matter proceeding from another individual, or from the inflamed eye. Others assert that the presence alone of a bougie is sufficient to give rise to the discharge. There are, it is true, facts which tend to prove that on producing inflammation of the urethra, the intensity of the ocular inflammation has been diminished; but I must observe to you, that this plan of treatment can only be indicated when the gonorrhœal conjunctivitis is metastatic, that is, when it has occurred immediately after the decrease or the suppression of the urethral discharge. Again, prudence will scarcely, in my opinion, allow us to attack a disease so rapid in its progress by a treatment the action of which is comparatively slow.

The same theoretical views have induced some surgeons to employ the internal remedies which are used in gonorrhœa. The objections I have just made may be repeated here. The action of these medicinal agents is too slow for it to be prudent to employ them in the first or acute period of the disease; when the inflammation has in a great measure subsided, and then only advantage may be derived from this method of treatment. In cases of subacute inflammation, I have myself found cubebs and the balsam of copaiba, given in large doses, extremely beneficial.

The treatment I shall definitively recommend to you may be resumed in a few words. Copious bleeding should be resorted to during the first period of the disease, until the inflammatory symptoms are entirely subdued. Astringent collyria may then be applied to the inflamed parts, and a revulsive action set up in the intestinal canal. When the disease is to be attributed to metastasis, one of the methods I have described may be employed, with the view of recalling the urethral discharge.

By thus uniting general and local treatment, I have succeeded in all the cases I have treated, when the cornea was not already affected at the time they came under my care.

When the inflammatory symptoms have abated, the disease may yet prove more or less rebellious to the remedies you employ; you need not, however, entertain any fear respecting the sight of the patient. In many cases granulations of variable size remain on the conjunctiva, and resist the action of all astringent collyria, none of which seem sufficiently energetic to destroy them. Recourse must then be had to cauterization with the solid nitrate of silver; and should it likewise fail, to excision of the granulated portion of the conjunctiva. I would, however, advise you only to adopt this last plan of treatment when all other means have failed, as excision of a portion of the conjunctiva is sometimes attended with very unfortunate results.

*The Egyptian Purulent Conjunctivitis.*

On comparing the published descriptions of the epidemics of purulent ophthalmia which have been observed in various countries, I have found so much analogy between them and the Egyptian form of purulent inflammation, that I do not hesitate to describe them under the same head. If these descriptions are faithful, as, indeed, I am inclined to believe they are, the morbid symptoms are nearly always the same, as also the treatment. I cannot, therefore, see any really valid reason for treating separately of each of these affections. It appears, however, that the Belgian epidemic presents some symptoms which have not been noticed in other epidemics of the same nature. Thus it has been asserted that granulations are to be met with on the conjunctiva, and that these granulations have not been observed before in purulent conjunctivitis. Is it, however, certain that granulations did not exist in the Egyptian ophthalmia? Is it certain that they were not then overlooked by our surgeons, who do not, it is true, mention their presence? This is a question which deserves to be investigated; I myself am inclined to think that they exist in all cases of purulent conjunctivitis. We will now examine what are the characters of this disease, and you will then, I hope, have a clear idea of the various purulent forms of inflammation.

The contagious or non-contagious nature of the Egyptian purulent form of inflammation has been the subject of much discussion. Most persons now agree in considering it to be contagious; and this opinion, which is substantiated by numerous well-authenticated facts, has not been shaken by the experiments performed on himself by Mackely, and quoted by Mackenzie in his treatise on Diseases of the Eye, page 184.

The etiology of this affection is extremely obscure. In Egypt, the reflection from the sand, the nature of the climate, &c., are quite sufficient to account for its presence. When, however, we find it appearing on the transports between Leghorn and Egypt—when we see it exercising its ravages in other countries, in England, in Belgium, and that with the same violence as in the country where it was first observed—we can no longer attribute its existence to these causes alone. If we admit the contagious nature of the malady, we can easily account for its attacking troops on their passage to Egypt; it is not, however, equally easy to prove that the epidemics, to which I have alluded, have been imported from that country, as some writers have asserted.

Other reasons have been given to account for the appearance of the disease. Thus the Belgium epidemic is attributed by M. Vleminckx to the soldiers wearing *schakos*, which are too heavy, to their washing their heads with cold water, and to the collar, which forms part of their uniform, being too tight, and consequently compressing the neck. These causes are not, most certainly, worthy of the attention they have met with. How is it, indeed, that the malady has only existed in the Belgian army for the last six or seven years, if its existence is to be explained by causes which have always been in action? M. Vanhonsbrooch is not much more rational in his attempt to explain the origin of the epidemic, when he attributes it to the irritating action on the eyes of the subcarbonate of lime, which is used by the soldiers in cleaning their buffletery. Would it not be better to acknowledge at once, that in this instance, as in many others, we are unacquainted with the primary cause of the disease?

The form of purulent inflammation is similar, in many respects, to gonorrhœal conjunctivitis. It presents the same rapidity in the appearance of the symptoms, the same intensity, and is followed by nearly the same consequences. In the disease we are examining, however, the inflammation generally attacks both eyes at once, although, in some instances, one of them is affected some time before the other. In gonorrhœal conjunctivitis, on the contrary, generally speaking, one eye only is the seat of disease. When the progress of the malady is not too rapid to allow us to observe the various symptoms, we find they manifest themselves in the following order:—

The patient complains at first of intense itching in the eye: this symptom generally makes its appearance in the evening. The itching is soon followed by the peculiar sensation of which we have so often spoken—that of extraneous bodies placed between the eye and the mucous surface of the palpebræ. The conjunctiva at the same time becomes inflamed; its tissue, as also the cellular lamellæ on the internal surface, are gorged with fluid, and tumefied. The vascularization of the conjunctiva is from the commencement extremely well defined; the size of the caruncula lacrymalis is much increased. The parts thus tumefied are soft, rather elastic, and bleed easily. This is not, however, an unfavourable circumstance, as the slight hæmorrhage which is thus produced tends to diminish the swelling. Four-and-twenty hours after the appearance of the above symptoms, a slightly viscid and opaque mucus is secreted, and the inflammation then extends to the entire internal surface of the palpebræ. This mucus soon assumes a thick, yellowish, purulent appearance. The eyelids become very much tumefied, especially the upper eyelid, which often covers nearly entirely the lower one. When they are separated, a considerable quantity of the purulent secretion escapes from the eye, and, flowing over the face, excoriates the skin wherever it passes. The ocular conjunctiva may become of a fungous nature, and be swollen to such an extent as to separate the free edges of the eyelids, and protrude from between them. When this is the case, the conjunctiva being irritated by the contact of the air, the secretion is extremely abundant. It may, indeed, according to Dr. Vetch, amount to several ounces daily.

Such are the principal symptoms of the Egyptian purulent conjunctivitis. Unfortunately this disease terminates, in most cases, by the extension of the inflammation to the cornea, and the total loss of the eye. As we have examined this complication, when speaking of gonorrhœal conjunctivitis, I shall refer you to what I then said on the subject.

Besides the local symptoms which I have just described, there are also general symptoms, which must not be omitted. The pulse is small, sometimes hard; generally, however, it remains soft. The skin is hot; the appetite nearly always remains good. Should the disease continue for several months, the patient generally falls a victim to diarrhœa, dysentery, and marasmus.

I shall not enter into any details respecting the treatment of this disease. It is in every respect similar to that of gonorrhœal conjunctivitis, with which we are already acquainted; with the exception, however, of those indications which are founded on the gonorrhœal nature of the disease.—*London Medical Gazette.*

